# Draft Genome Sequence of Deinococcus koreensis SJW1-2^T^, a Gamma Radiation-Resistant Bacterium Isolated from River Water

**DOI:** 10.1128/MRA.00894-18

**Published:** 2018-08-16

**Authors:** Kiwoon Baek, Eu Jin Chung, Gang-Guk Choi, Young Ho Nam, Ahyoung Choi

**Affiliations:** a Freshwater Bioresources Research Bureau, Nakdonggang National Institute of Biological Resources (NNIBR), Sangju, South Korea; b Freshwater Bioresources Culture Research Bureau, Nakdonggang National Institute of Biological Resources (NNIBR), Sangju, South Korea; Queens College

## Abstract

Deinococcus koreensis SJW1-2^T^ was isolated from river water and was observed to be highly resistant to gamma radiation. In this study, we report a draft genome sequence which revealed that SJW1-2^T^ possesses genes involved in nucleotide excision repair. The primary genomic information will aid in elucidating the DNA repair mechanism during ionizing radiation.

## Abstract (precis)

Deinococcus koreensis SJW1-2^T^ was isolated from river water and was observed to be highly resistant to gamma radiation. In this study, we report a draft genome sequence which revealed that SJW1-2^T^ possesses genes involved in nucleotide excision repair.

## ANNOUNCEMENT

The genus Deinococcus belongs to the family Deinococcaceae. Deinococcus radiodurans, the type species of this genus, was isolated from canned meat that had spoiled following exposure to X rays (1). Currently, more than 70 species of this genus have been published with a valid name (http://www.bacterio.net). The genus Deinococcus comprises mainly Gram-positive (some are Gram negative) aerobic coccus- or rod-shaped bacteria that are generally resistant to gamma or UV radiation. Here, we report the draft genome sequence of Deinococcus koreensis SJW1-2^T^ (=KACC 19332 = NBRC 112908) isolated from Seomjin River in South Korea (N35°03ʹ48.2ʺ E127°44ʹ29.2ʺ). The bacterium is aerobic, Gram negative, rod shaped, nonmotile, and pink pigmented, with optimum growth at 25°C. Strain SJW1-2^T^ is resistant to gamma radiation of 10.6 kilograys (kGy) (2). It is closely related to Deinococcus metalli 1PNM-19^T^, with 94.3% similarity in the 16S rRNA gene.

For the genome sequence of strain SJW1-2^T^, genomic DNA was extracted using a DNeasy blood and tissue kit (Qiagen, Valencia, CA, USA). A 20-kb-insert SMRTbell genomic DNA library was sequenced by Macrogen (Seoul, South Korea) using the PacBio RS II platform (Pacific Biosciences, Menlo Park, CA, USA). The bacterial genome was assembled *de novo* into six contigs, with an average coverage of 171.17×, using the PacBio SMRT Portal (2.3.0) and the Hierarchical Genome Assembly Process (3). The data were submitted to the Rapid Annotations using Subsystems Technology server (4) and the National Center for Biotechnology Information genome sequence database. Potential coding sequences were searched for using the Basic Local Alignment Search Tool against the UniProt (5), Pfam (6), and Clusters of Orthologous Groups (7) databases. Signal peptides and transmembrane helices were predicted using SignalP 4.1 (8) and TMHMM version 2.0 (9). RNAs and other miscellaneous features were predicted using RNAmmer version 1.2 (10), tRNAscan-SE version 1.21 (11), and Rfam version 12.0 (12), respectively. Automatic detection of clustered regularly interspaced short palindromic repeats was conducted using MinCED version 0.2.0 (13).

The genome consists of six contigs, with a G+C content of 69.5% and a genome size of 4,500,921 bp. In total, 4,085 coding sequences were identified, which accounted for 87.37% of the genome. Additionally, the genome contains 58 tRNA genes and 9 rRNA genes. The genome of D. koreensis SJW1-2^T^, similar to that of Deinococcus gobiensis, contains 4 genes coding for Lon protease (SJW1-2_00304, SJW1-2_01038, SJW1-2_01083, and SJW1-2_01874), 1 proteolytic ClpP subunit (SJW1-2_01876), and 6 ATPase subunits found in ClpA (SJW1-2_00092, SJW1-2_00254, and SJW1-2_00280), ClpB (SJW1-2_03236), ClpS (SJW1-2_00279), and ClpX (SJW1-2_01875). Lon proteases and Clp are involved in the protection against gamma irradiation via the repair of DNA double-strand breaks (14).

SJW1-2^T^ was previously shown to be resistant to gamma radiation. The genome data reported here provide details regarding the potential mechanism, involving nucleotide excision repair.

### Data availability.

The genome sequence of Deinococcus koreensis SJW1-2^T^ has been deposited in GenBank under accession number PPPD00000000 (BioProject number PRJNA419019). The version described in this paper is the first version.
